# Magnetic resonance imaging -based radiomics of the pituitary gland is highly predictive of precocious puberty in girls: a pilot study

**DOI:** 10.3389/fendo.2025.1496554

**Published:** 2025-02-05

**Authors:** Michele Maddalo, Maddalena Petraroli, Francesca Ormitti, Alice Fulgoni, Margherita Gnocchi, Marco Masetti, Eugenia Borgia, Benedetta Piccolo, Emanuela C. Turco, Viviana D. Patianna, Nicola Sverzellati, Susanna Esposito, Caterina Ghetti, Maria E. Street

**Affiliations:** ^1^ Medical Physics Department, University Hospital of Parma, Parma, Italy; ^2^ Unit of Pediatrics, Department of Mother and Child, University Hospital of Parma, Parma, Italy; ^3^ Neuroradiology Unit, University Hospital of Parma, Parma, Italy; ^4^ Department of Medicine and Surgery, University of Parma, Parma, Italy

**Keywords:** central precocious puberty, pituitary gland, radiomics, machine learning, magnetic resonance imaging, precocious puberty, puberty

## Abstract

**Background:**

The aim of the study was to explore a radiomic model that could assist physicians in the diagnosis of central precocious puberty (CPP). A predictive model based on radiomic features (RFs), extracted form magnetic resonance imaging (MRI) of the pituitary gland, was thus developed to distinguish between CPP and control subjects.

**Methods:**

45 girls with confirmed diagnosis of CPP (CA:8.4 ± 0.9 yr) according to the current criteria and 47 age-matched pre-pubertal control subjects (CA:8.7 ± 1.2 yr) were retrospectively enrolled. Two readers (R1, R2) blindly segmented the pituitary gland on MRI studies for RFs and performed a manual estimation of the pituitary volume. Radiomics was compared against pituitary volume in terms of predictive performances (metrics: ROC-AUC, accuracy, sensitivity and specificity) and reliability (metric: intraclass correlation coefficient, ICC). Pearson correlation between RFs and auxological, biochemical, and ultrasound data was also computed.

**Results:**

Two different radiomic parameters, Shape Surface Volume Ratio and Glrlm Gray Level Non-Uniformity, predicted CPP with a high diagnostic accuracy (ROC-AUC 0.81 ± 0.08) through the application of our ML algorithm. Anthropometric variables were not confounding factors of these RFs suggesting that premature thelarche and/or pubarche would not be potentially misclassified. The selected RFs correlated with baseline and peak LH (p < 0.05) after GnRH stimulation. The diagnostic sensitivity was improved compared to pituitary volume only (0.76 versus 0.68, p<0.001) and demonstrated higher inter-reader reliability (ICC>0.57 versus ICC=0.46).

**Discussion:**

Radiomics is a promising tool to diagnose CPP as it reflects also functional aspects. Further studies are warranted to validate these preliminary data.

## 1 Introduction

Central precocious puberty (CPP) is a condition of premature activation of the hypothalamus-pituitary-gonadal (HPG) axis (1). The key players involved are regulators of the hypothalamus that secretes gonadotropin-releasing hormone (GnRH) which stimulates in turn the anterior pituitary gland to secrete LH and FSH; these then trigger the production of sexual hormones by the gonads (2, 3).

Currently, the diagnosis of CPP relies on the gonadotropin-releasing hormone (GnRH) stimulation test (4, 5) in addition to clinical, and radiological data (6). Among these latter the ultrasonographic evaluation of the internal genitalia in females allows the exclusion of malignancies or cystic lesions and can confirm ovarian activation and hormonal stimulation of the uterus (7). Magnetic resonance imaging (MRI) of the brain and of the hypothalamus-pituitary region is required to exclude central organic causes. At present, the general advice is to perform MRI in all girls under the age of 8 yr and all boys under 9 yr with CPP (8, 9). The overall prevalence of unsuspected intracranial lesions is reported to be 5,7-40% in boys, and 8-33% in girls (10, 11). The estimated risk of detecting a tumor in girls between the ages of 6 and 8 yr is low (12), therefore, it is debated whether this screening should be performed or not above the age of 6 yr. A recent study on a cohort of 112 girls showed that hypothalamic-pituitary congenital/developmental anomalies and other cerebral lesions were present in less than half of the subjects (13). Thus, as evidence-based criteria are yet lacking, brain MRI is currently recommended (7, 14).

During pubertal development the pituitary gland undergoes changes in shape and volume. Typically, a higher pituitary grade, height, and sagittal cross-sectional area are observed (14). Subsequently the pituitary gland becomes more convex superiorly, with a significantly lower length/height index making the finding of a nearly spherical gland in association with early sexual development particularly suggestive of CPP (15). It has been reported that pituitary volume is increased in children with CPP (16) and some authors have suggested that volume per se might be predictive of CPP reporting a sensitivity of 0.54, and a specificity 0.72. Moreover, pituitary volume was described to be associated also with LH serum concentrations, LH/FSH ratio and bone age although a reliable cut-off value was not found (17). Sex steroids levels have been described also to be positively associated with the volume of the pituitary gland (18).

To date artificial Intelligence (AI) includes image analysis and related algorithms that fall in the category of Computer Vision (CV). Image segmentation represents a fundamental technique in CV allowing image processing to extract meaningful information from digital images and helping to find diagnostic and decision making-algorithms (19). In medicine, the term that technically defines this new methodologic approach is radiomics, an emerging research field holding enormous potential (20).

Radiomics can be considered the bridge between medical imaging and personalized medicine, as its main goal is to correlate large numbers of quantitative parameters with clinical or biological endpoints, thus supporting clinical decisions, in the context of diagnostic processes, prognostic evaluation and in the analysis of therapeutic response (21). Furthermore, radiomics enables the identification of potentially important information that may not be appreciable upon visual examination (22). Deep Learning networks have significantly improved the ability of image segmentation, specifically through the application of Convolutional Neural Networks (CNNs), that consume pixels of images as input and are able to recognize patterns in the image itself (23). CNNs can be taught to predict the features of interest from novel medical datasets.

This study aimed at analyzing through a radiomic model, MRI scans of the pituitary gland, in girls with CPP compared with a pre-pubertal age and sex-matched control group to study the differences in information compared with the traditional method. Moreover, any possible associations among auxological, hormonal and radiological parameters and radiomic data were investigated to better understand any relationships with morphological features of the pituitary gland and the clinical and hormonal presentation of CPP for diagnostic purposes.

## 2 Materials and methods

### 2.1 Study design and subjects

The MRI scans of 45 girls (mean age ± SD at diagnosis: 8.4 ± 0.9 yr) referred for precocious pubertal development to the Pediatric Endocrinology Unit of the Children’s University Hospital in Parma (Italy) from January 2015 to December 2022, who received a conclusive diagnosis of CPP (4) were analyzed, and compared with the findings of 47 pre-pubertal age-matched girls (mean age± SD: 8.7 ± 1.2 yr) who were referred to the Pediatric Neurology Unit of the same hospital and that underwent brain MRI for headaches, brain trauma, epilepsy and nonspecific neurological symptoms with negative findings. Exclusion criteria were: male sex, non-idiopathic CPP including premature thelarche, presence of significant comorbidities or other endocrine conditions requiring hormonal therapies or any medications that may interfere with pubertal development.

All subjects were diagnosed of idiopathic CPP with no evidence of hypothalamic-pituitary congenital malformations, other endocrine or chronic diseases, neurological disorders, or malignancies. As to ethnicity, 3 girls were African and 2 Indian, 4 girls of non-Italian origin were adopted and family history and mid-parental height were unavailable. The remaining were Caucasians. At the time of the first assessment, all patients underwent a clinical examination including an auxological evaluation. Pubertal stages were assessed according to Tanner’s criteria (24). Height was recorded using a Harpenden stadiometer and body mass index (BMI) was calculated according to the formula weight (kg)/height2 (m2), both expressed as standard deviation scores (SDS) using the Italian reference data (25). Target height (THt) was calculated based on sex-adjusted mid-parental height [father’s height + mother’s height –13]/2 and converted to SDS (26). Height, BMI and THt were plotted on Italian reference growth charts by Cacciari et al. (27).

Medical history, family history of precocious puberty, parents’ height, and age at the onset of pubertal signs were recorded. Moreover, the rate of pubertal progression, defined as the time elapsed between the appearance of Tanner breast stage 2 (as recorded by the general pediatrician or referred by parents) and diagnosis was also evaluated. Bone age was assessed using the Greulich & Pyle Atlas (28). Bone age advancement was defined as the difference between bone age and chronological age.

Pelvic trans-abdominal ultrasound was performed to assess the degree of maturation of internal genitalia. Uterine length, the fundus/cervix ratio, the ovarian volumes, and the presence or absence of endometrial thickening were evaluated. A uterine longitudinal diameter ≥ 35 mm, a body/cervix ratio ≥ 1, an ovarian volume of ≥ 2 ml, and the presence of endometrial thickening were considered suggestive of pubertal activation (29–31).

GnRH stimulation test was performed by intra venous administration of GnRH at a dose of 75 μg/m2 (maximum 100 μg), with measurement of LH and FSH by chemiluminescent immune assays (Beckman Coulter) at times 0, + 15, + 30, + 45, + 60, + 90 minutes. We considered peak LH and FSH above 5 IU/L suggestive of CPP (4). Only in one girl the GnRH test was not performed due to significant increased basal gonadotropin levels. For all patients basal estradiol was recorded, and thyroid function (TSH, FT4) was normal in all subjects.

The diagnosis of CPP was based on age, pubertal stages, LH and FSH response to the GnRH test, pelvic US and bone age.

Clinical, hormonal and pelvic ultrasound findings of the girls having CPP are reported in **Table 1**.

**Table 1:** Clinical, hormonal and pelvic ultrasound findings of the girls diagnosed of CPP.

Clinical Parameter	Mean ± SD
CA (years)	8.2 ± 0.6
Bone Age (years)	9.5 ± 1.2
BMI SDS	-0.2 ± 1.0
Height SDS	1.0 ± 0.9
Hormonal measurements	Mean ± SD
Basal LH (mU/mL)	1.6 ± 1.4
basal FSH (mU/mL)	4.7 ± 2.2
peak LH (mU/mL)	23.7 ± 16.0
peak FSH (mU/mL)	14.5 ± 4.6
Δ LH	22.2 ± 15.2
Δ FSH	9.6 ± 4.5
TSH (uUI/mL)	2.1 ± 1.6
FT4 (ng/dL)	0.9 ± 0.1
Ultrasonographic parameters	Mean ± SD
Uterine body length (mm)	46.3 ± 8.1
Left ovarian volume (mL)	2.7 ± 1.5
Right ovarian volume (mL)	2.6 ± 1.2
Fundus/cervix ratio	1.5 ± 0.6

Data are reported as mean values and associated standard deviations (SD). Regarding Hormonal measurements, peak hormonal values were acquired after GnRH stimulation and Δ values represented the differences between peak and basal LH and FSH values.

### 2.2 Magnetic resonance imaging of the pituitary gland and segmentation

MRI was performed on a 1.5-T MRI (1.5T Philips Ingenia, Best, The Netherlands). The sagittal T1W3D TFE sequence with no contrast agents was selected to perform image segmentation of the pituitary gland. Repetition time and echo time were equal to 8.1 ms and 3.7 ms respectively, while the slice thickness was set to 1 mm.

The pituitary volume (PV) was calculated using the ellipsoid formula L*H*W/2 (17, 32, 33). Length and height were determined on the midline sagittal thin section from the posterior wall to the anterior wall. The width was measured on the thin coronal section from anterior to the entrance of the pituitary stalk.

MR images were independently and blindly reviewed by two readers with different levels of expertise, i.e. a neuroradiologist with 4-years of experience (R1) and a 1-year radiology resident (R2). Both readers manually evaluated PV (ellipsoid formula) and performed the segmentation of the pituitary gland using the 3D Slicer v. 5.0.3 software (34).

### 2.3 Radiomics

The extraction of radiomics features was carried out using the open-source python package Pyradiomics (35). An isotropic voxel size of 1 x 1 x 1 mm was considered in order to avoid that Radiomic Features (RFs) could depend on the image sampling condition.

We included only original features, without performing image preprocessing, thus, the extracted RFs included both first-order and subsequent-order features including shape, first-order, Gray-Level-Co-occurrence-Matrix (Glcm), Gray-Level-Run-Length-Matrix (Glrlm), Gray-Level-Size-Zone-Matrix (Glszm), Neighboring-Gray-Tone-Difference-Matrix (Ngtdm) and Gray-Level-Dependence-Matrix (Gldm) functions, for a total of 107 extracted RFs.

### 2.4 Machine learning and statistical analysis

A Monte Carlo Cross-Validation (MCCV) test with 100 rounds was implemented considering a proportion of 80%-20% and balancing with respect to the endpoint. MCCV iteratively performed a random split of the database into training and validation sets with a different patient inclusion among rounds.

All the following analyses were cross validated using MCCV:

1. Radiomic model development.
2. Reference clinical model development.
3. Evaluation of the impact of confounders.
4. Association of radiomic features with endpoint surrogates.
5. Evaluation of reliability of both clinical and radiomic model parameters between R1 and R2.

#### 2.4.1 Radiomic model

The analysis pipeline included two distinct and consecutive phases, the first for feature selection and the second for training and validation of the classifier.

The first phase was performed 100 times on the training set. Patients’ inclusion in the training set was iteratively modified using a random approach (i.e. the MCCV). This phase consisted of the following operations:

1. Removal of highly cross-correlated RFs. A Pearson correlation coefficient greater than 0.99 was considered indicative of redundancy.
2. Evaluation of RF predicting capability using univariable Mann-Whitney nonparametric test. In each round, RFs with a p-value below 0.001 were scored.

At the end of 100 rounds the top two RFs with the greatest cumulative score (sum of scores across rounds) were selected. The selected RFs were not included in the subset of RFs that correlated with at least one of the confounders.

In the second phase a MCCV with 100 training/validation splits was implemented again. A Linear Discriminant Analysis (LDA) (36, 37) classifier was trained on training data using only the two top- scored RFs. Optimal thresholds for the selected RFs were identified using the Youden method for comparison between readers. In each round, the trained models were then applied without any modification on the validation set for unbiased evaluation of the model performances. Validation performances were thus determined. Following the described methodology, two distinct models were developed, one based on data generated by R1 and another one based on data from R2. The R1 radiomic model was considered as the primary, while the R2 model was added for comparison to understand the variability of results and the reliability of the algorithms with respect to reader’s expertise. Furthermore, top-scored RFs yielded by R1 segmentation were employed for R2 model development also.

Performance metrics included ROC-AUC, accuracy, sensitivity, specificity and G-Mean (the square root of the product of sensitivity and specificity) and were expressed as mean values of 100 iterations with associated standard deviations. Mean threshold values and their ranges across 100 rounds were calculated. The consistency of findings between R1 and R2 was evaluated by calculating the absolute difference between R1 and R2 mean performances. A cumulative value that aggregated all performance metrics was computed by adding together all metrics differences. The analysis pipeline was developed using the R software environment (38) with Caret (39) and MASS (40) packages.

#### 2.4.2 Reference model

The PVs were used to develop a reference model for comparison purposes. A single predictor was considered. The same methodology as described for radiomics (second phase) was used to develop the reference model. One reference model per each reader was produced. The correlation between the manual evaluation of PV and the PV obtained by radiomics was tested for consistency purposes.

#### 2.4.3 Evaluation of the impact of possible confounders

Age, height, BMI and bone age of patients were tested as possible confounding parameters for radiomic predictors and for the pituitary volume (ellipsoid formula). These were continuous variables, so Pearson correlation was used to assess the association of RFs with each possible confounder parameter. Correlations were considered as statistically significant only if the p-value (on training data) was less than 0.05 in at least 50% of the rounds of the MCCV. The mean value of the correlation coefficient (®R) across 100 rounds was then calculated.

#### 2.4.4 Association analyses of RFs with clinical, biochemical and pelvic ultrasound data

The present analysis was conducted on the CPP patients only.

Endpoint surrogates included both categorical (ordered) variables, i.e. pubertal stages, and continuous variables, i.e. basal and peak LH, and FSH, estradiol, ovarian volumes, uterine length, and fundus/cervix ratio.

The association of RFs with categorical endpoint surrogates was evaluated by means of the Kruskal-Wallis nonparametric test. As for the evaluation of confounders, in each round of MCCV, the RFs that showed a p-value (on training data) below 0.05 were tracked. At the end of the MCCV procedure the RFs with a cumulative score greater than 50% were considered significantly associated with the endpoint surrogate. Pearson correlation was computed among RFs and continuous endpoint surrogates. To evaluate correlations, the same approach was used for confounders calculations of R and p-values in each repetition on the training set, while the final classification was established based on cumulative scores over 100 rounds.

#### 2.4.5 Evaluation of reliability between R1 and R2

Reliability of RFs yielded by R1 and R2 segmentations was evaluated using the Intraclass Correlation Coefficient (ICC). The reliability of the manual evaluation of PV was assessed in addition. The ICC was computed by a single-rating, absolute-agreement, 2-way random-effect model and then the level of agreement was evaluated using the following general guideline (41): values less than 0.5 are suggestive of poor reliability, between 0.5 and 0.75 indicate moderate reliability, between 0.75 and 0.9 indicate good reliability, and greater than 0.90 indicate excellent reliability.

## 3 Results

### 3.1 Machine learning and statistical analysis

#### 3.1.1 Radiomic and reference models

**Figure 1:**
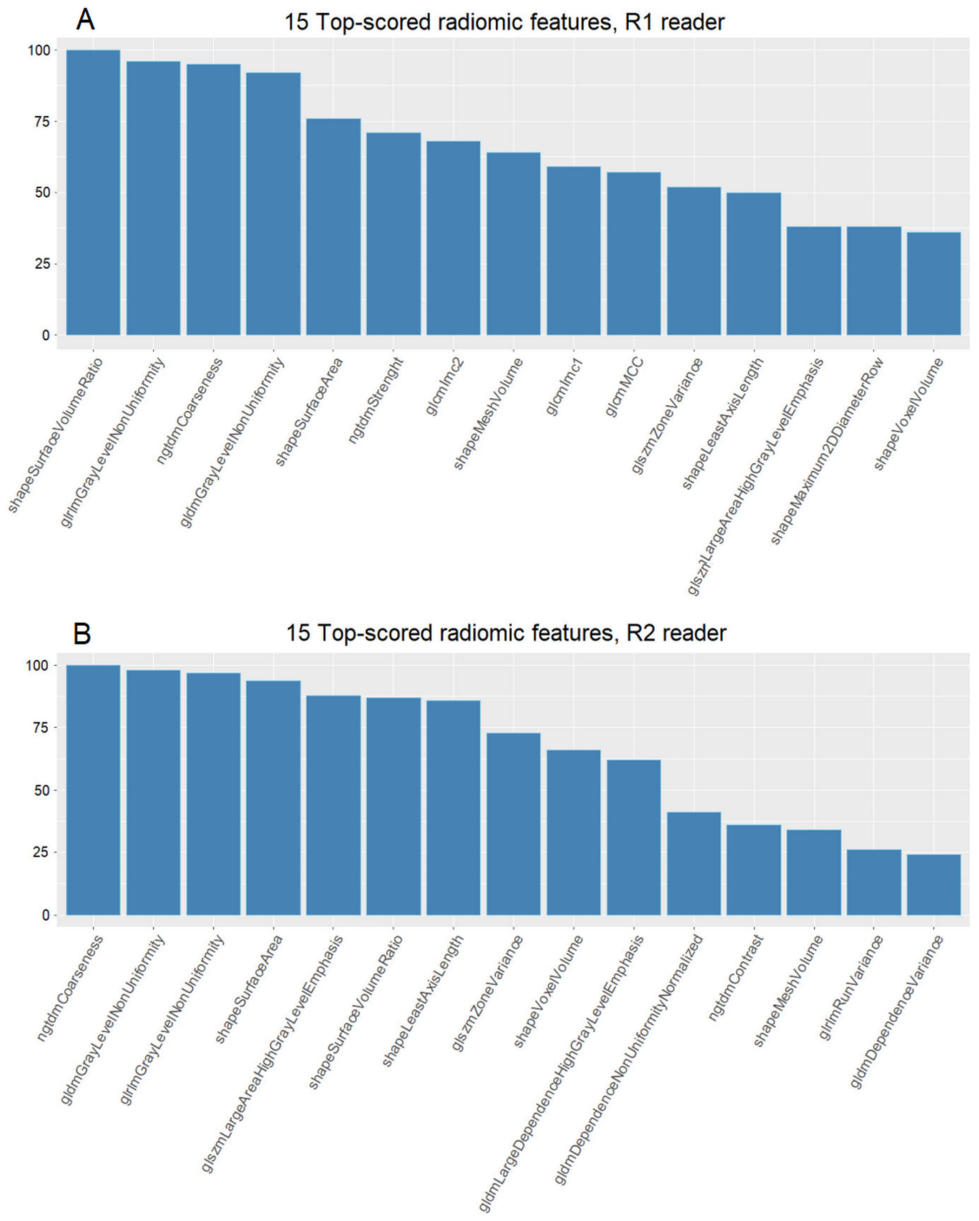
Cumulative scores of the top 15 radiomic features, calculated as the sum of scores obtained over 100 rounds. **(A) **Feature scores derived from R1 segmentation and **(B)** feature scores obtained from R2 segmentation.

The top scored RFs after 100 rounds of feature selection are reported in **Figures 1A, B**, for R1 and R2 readers, respectively. The two radiomic predictors with the greatest predictive capability for CPP for R1 were: Surface Volume Ratio (shape feature) and Gray Level Non-Uniformity from Glrlm matrix. Surface Volume Ratio represents a shape parameter and gives morphologic information, evaluating the degree of sphericity of the gland. Conversely, Gray Level Non-Uniformity is a second-order parameter that measures the difference in gray levels within the pituitary gland: the higher, the more the voxels show different intensity, thus evaluating the non-uniformity of grey levels. The predictors with the highest predictivity for R2 were Ngtdm Coarseness and Gray Level Non-Uniformity of GLDM matrix. Shape Surface Volume Ratio and Glrlm Gray Level Non-Uniformity was also highly ranked (positions 3 and 6, respectively) (**Figure 1B**).

Mean and standard deviations of the two top-scored RFs and corresponding pituitary volumes (ellipsoid formula) are listed in **Table 2** for both readers.

**Table 2:** Mean values of radiomic and clinical predictors, and thresholds of predictive models.

		Shape Surface Volume Ratio	Glrlm Gray Level Non-Uniformity	Volume (ellipsoid method)
Whole population	R1	1.14 ± 0.22	44.7 ± 16.5	0.20 ± 0.09
	R2	1.08 ± 0.20	48.8 ± 17.4	0.23 ± 0.08
Positive class (CCPs)	R1	1.05 ± 0.17	52.5 ± 16.7	0.25 ± 0.08
	R2	1.01 ± 0.14	56.1 ± 17.5	0.26 ± 0.09
Negative class (controls)	R1	1.23 ± 0.22	37.2 ± 12.4	0.16 ± 0.06
	R2	1.15 ± 0.22	41.7 ± 14.2	0.20 ± 0.07
Threshold (Youden method)	R1	1.13 ± 0.0	46.9 ± 4.0	0.21 ± 0.01
	R2	1.02 ± 0.05	49.4 ± 2.5	0.26 ± 0.03

CPP patients and control subjects aggregated data were calculated. CPP cases presented a lower Surface Volume Ratio (i.e. a greater sphericity) and a higher GrayL Level Non-Uniformity (i.e. a lesser homogeneity of voxels intensities) with respect to control subjects.

Optimal thresholds (mean values and ranges) are reported in **Table 2** with good agreements between the two readers.

Significant correlations between manual and radiomic evaluations of the PV (**Supplementary Figure 1**) were observed for both R1 (R = 0.67, p < 0.001) and R2 (R = 0.71, p < 0.001).

**Figure 2:**
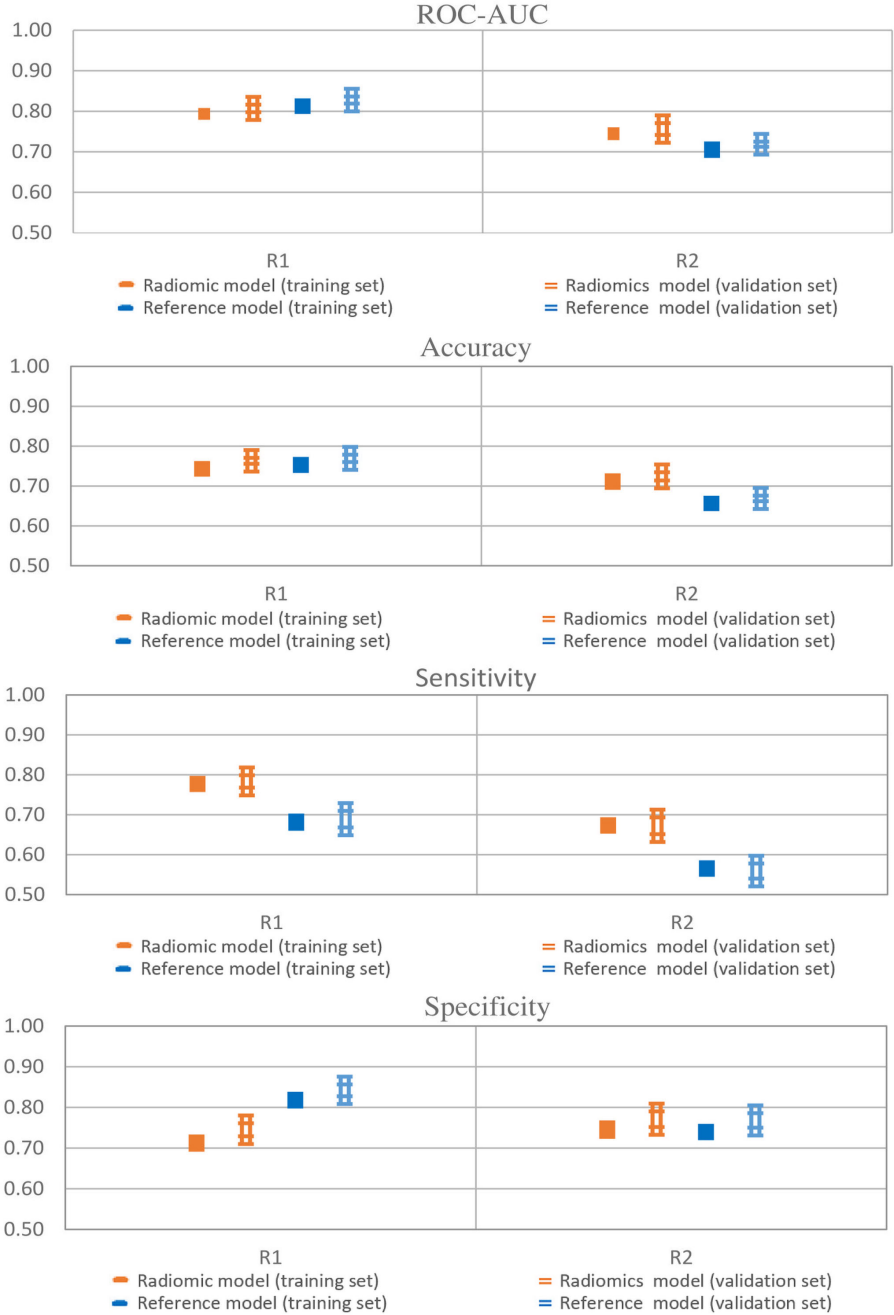
Model performances of radiomic and reference models. Error bars represent 95% confidence intervals. Single thick line: training set; Double thin lines: validation set; Orange: radiomics; Blue: Pituitary Volume using the ellipsoid method.

Performances of radiomic and reference models in training and validation sets are reported in **Figure 2** and showed a good agreement, indicating that no significant overfitting was present.

Radiomics achieved a ROC-AUC of 0.72–0.80 and an accuracy between 0.70 and 0.77, while the reference model showed a ROC-AUC between 0.72–0.83 and an accuracy of 0.67–0.77. The models showed differences in sensitivity and specificity. The sensitivity and specificity of the radiomics ranged from 0.67 to 0.76 and 0.72 to 0.76, respectively. In contrast, for the reference model sensitivity ranged from 0.56 to 0.69, and specificity ranged from 0.77 to 0.84. Therefore, the radiomic model achieved a well-balanced trade-off between sensitivity and specificity.

**Figure 3:**
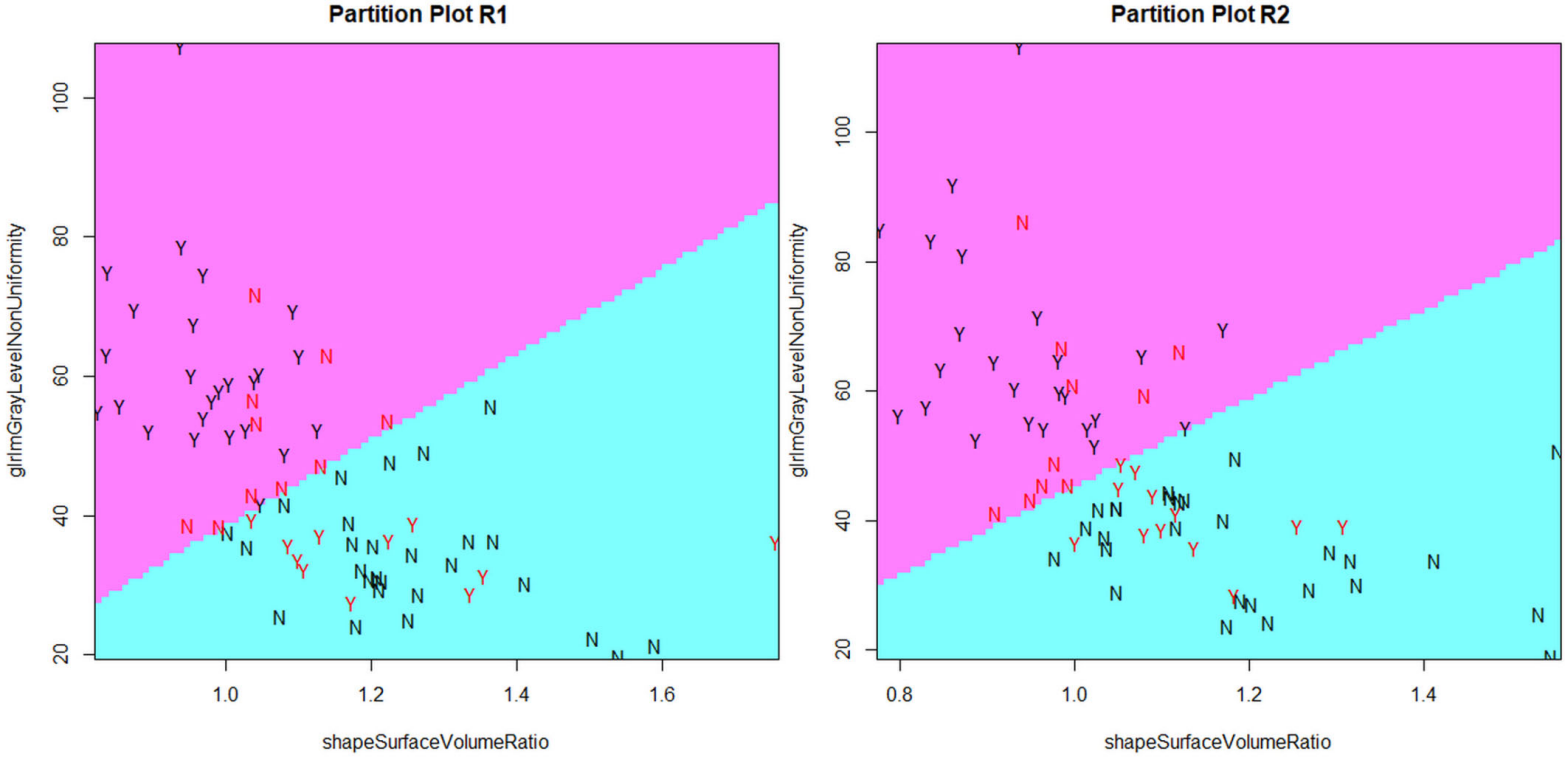
Scatter plots of training cases as determined by Surface Volume Ratio (Shape) and Gray Level Non-Uniformity (Glrlm) for both readers. The line that better discriminated the two classes is drawn. N (negative class): control cases; Y (positive class): CPP cases. Black cases denote correct predictions, while red cases are wrong predictions.

The scatter plot of training cases with the LDA line (CPP and controls) is reported in **Figure 3** for both readers with similar findings.

**Figure 4:**
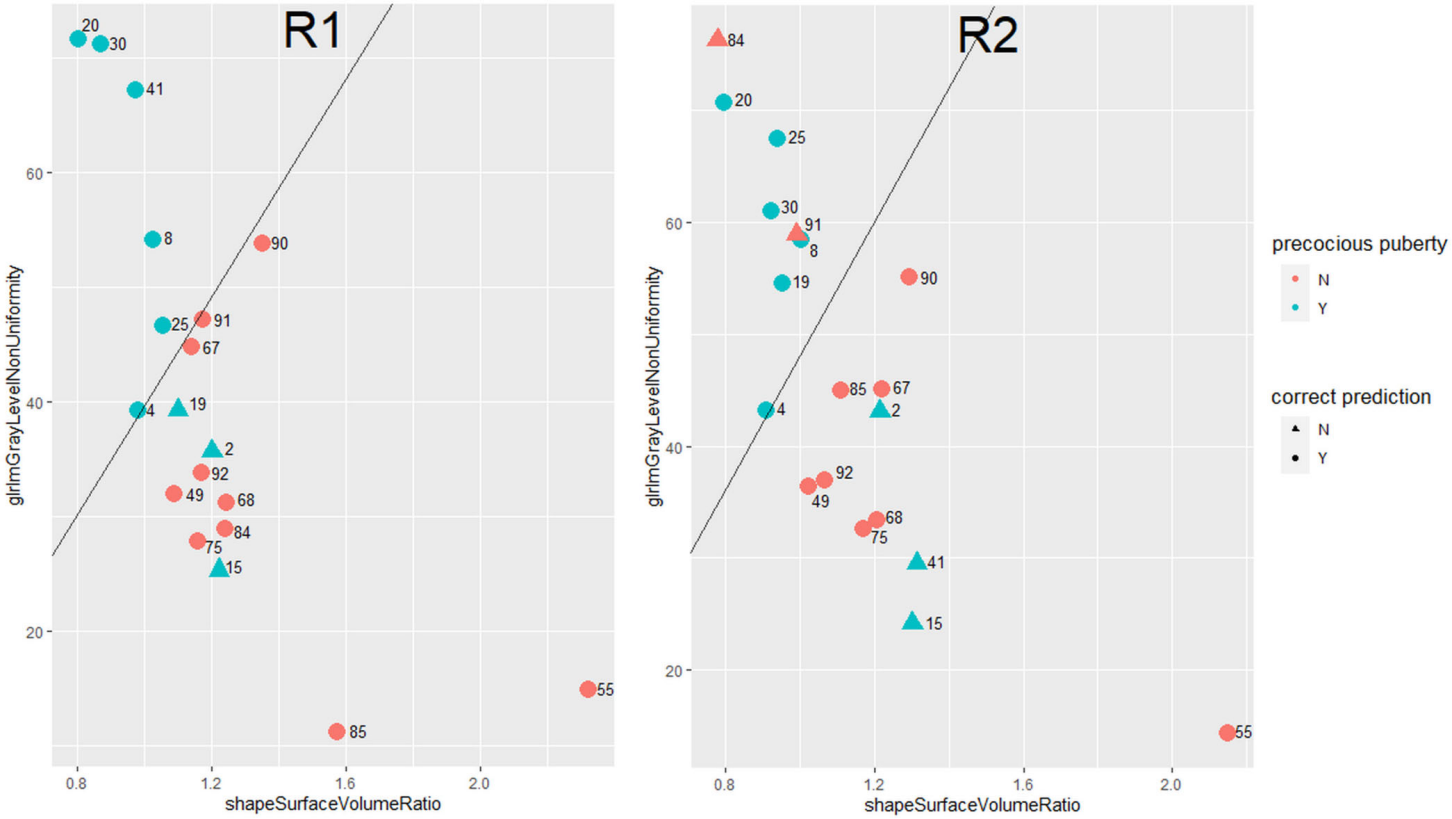
Scatter plot of validation cases as determined by Surface Volume Ratio (Shape) and Gray Level Non-Uniformity (Glrlm). Red color (negative class): control cases. Green color (positive class): CPP cases. Circle: correctly classified patients. Triangle: patients wrongly classified.

The scatter plot of validation cases with correctly/wrongly classified subjects is reported in **Figure 4** (round 49). Among the 19 validation cases, 74% had a compatible position in both R1 and R2 scattered plots. The remaining 26% of cases was not in agreement between R1 and R2, and a prediction error by one of the two models exists. Of these, four subjects were misclassified by one reader only, while two were misclassified by both.

**Figure 5:**
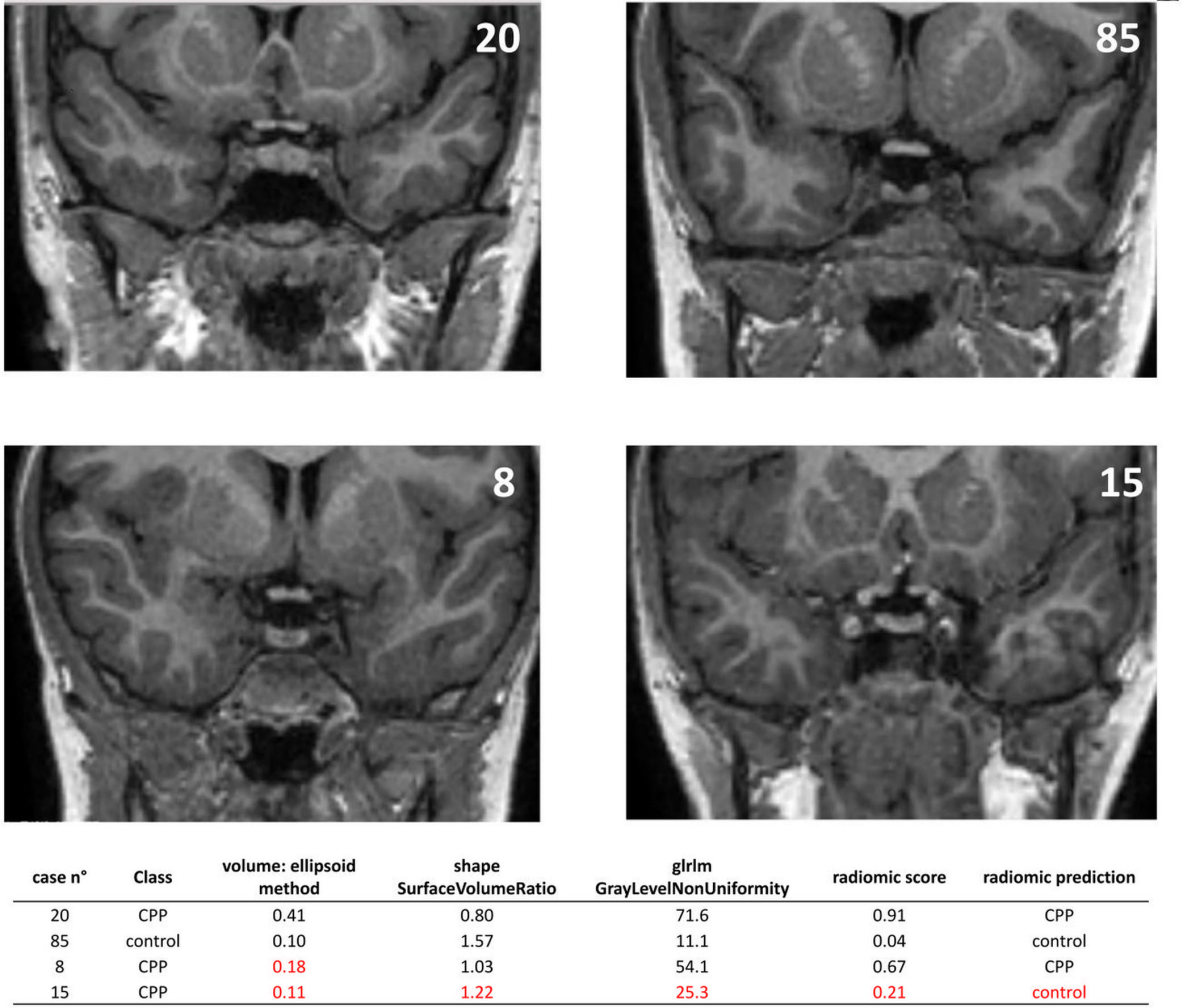
Graphical representation of four representative cases that help to interpret the classification mechanism of the developed predictive models.

The actual values of radiomic and clinical predictors were subsequently reported to a coronal MRI view of the corresponding subject for four randomly selected representative cases to better evaluate ML results (**Figure 5**). The first patient (n. 20) had a high pituitary volume, a more spherical shape and an accentuated non-homogeneity of voxel values and was successfully classified as CPP by both the clinical reference model and the radiomic model. Similarly, the second patient (n. 85) was correctly identified as a control, had a small pituitary volume, a flattened shape and a uniform texture of the pituitary gland. In the third patient (n. 8) texture data in addition to shape allowed the diagnosis of CPP. Finally, case n. 15 evidenced the possible limit of radiomics used alone but had a smaller pituitary volume, flattened shape and uniform texture.

#### 3.1.2 Evaluation of the impact of possible confounders

Correlations between RFs and confounders are reported in **Table 3**. Among the 107 RFs, 7 RFs were found to be modified by BMI, 3 by age, and 19 by bone age whereas none correlated with height. However, all observed correlations were weak (0.3≤|R ®|<0.5). Importantly, the selected RFs, Surface Volume Ratio (shape feature) and Gray Level Non-Uniformity (Glrlm) did not correlate with BMI SDS, height SDS, age, and bone age (**Table 3**). At variance, PV (ellipsoid formula) correlated with height SDS and bone age (R ® = 0.35, p < 0.05; R ® = 0.36; p < 0.05, respectively).

**Table 3:** Significant correlations between RFs and confounding parameters.

Confounder	RFs significantly correlated *	Size of correlation ^
BMI SDS	None	–
Height SDS	Volume: ellipsoid method	WEAK
Age	Volume: ellipsoid method	WEAK
Bone age	None	–

*Correlation with p-value < 0.05 in at least 50% of rounds.

^The size of the correlation was attributed according to the mean value of the correlation coefficient ( $\bar{\text{R}}$ ) across 100 rounds.

#### 3.1.3 Association analyses of RFs with clinical, biochemical and pelvic ultrasound data

Pubertal stages at diagnosis were not associated with the selected RFs, but with other non-shape radiomic features (**Table 4**).

**Table 4:** Significant associations between models’ predictors and categorical endpoint surrogates describing the clinical stage of puberty.

Endpoint surrogate	RFs significantly associated with the categorical endpoint surrogate*
Tanner Breast stage	Volume: ellipsoid method
Tanner Pubic Hair stage	None

*Significant difference (p-value < 0.05) of RF value among endpoint (surrogate) subgroups in at least 50% of rounds.

Correlations of RFs with hormonal levels and pelvic US features in girls with CPP are reported in **Table 5**. In detail, basal LH serum concentrations were correlated with the Shape Surface Volume Ratio (R ® = -0.44, p < 0.05) and the Glrlm Gray Level Non-Uniformity (R ® = 0.56, p < 0.05). Similarly, LH peak serum concentrations were correlated with the Shape Surface Volume Ratio (R ® = -0.45, p < 0.05) and Glrlm Gray Level Non-Uniformity (R ® = 0.49, p < 0.05). Associations with clinical, biochemical and pelvic ultrasound data of RFs that were not included in the radiomic model are reported in **Supplementary Tables 1** and **2**.

**Table 5:** Significant correlations between models’ predictors and hormonal and pelvic US endpoints.

Endpoint surrogates	RFs significantly correlated *	Size of correlation ^
basal LH	glrlmGrayLevelNonUniformity	MODERATE
	shapeSurfaceVolumeRatio	WEAK
	Volume: ellipsoid method	MODERATE
peak LH	glrlmGrayLevelNonUniformity	WEAK
	shapeSurfaceVolumeRatio	WEAK
basal FSH	Volume: ellipsoid method	WEAK
peak FSH	None	–
Left ovarian volume	glrlmGrayLevelNonUniformity	WEAK
Right ovarian volume	None	–
Uterine length	None	–
Fundus/cervix ratio	None	–
Estradiol	None	–

*Correlation with p-value < 0.05 in at least 50% of rounds. ^The size of correlation was attributed according to the mean value of the correlation coefficient ( $\bar{R}$ ) across 100 rounds.

#### 3.1.4 Evaluation of reliability between R1 and R2

Surface Volume Ratio and Gray Level Non-Uniformity had ICCs of 0.68 and 0.58, respectively for both radiomic predictors. The reliability of the predictor of the reference model was low (ICC = 0.47). Reliability data of all RFs for both R1 and R2 are reported in **Supplementary Table 3**.

## 4 Discussion

In this study we analysed radiomic data from MRI of the pituitary gland, extracted through image segmentation techniques, to investigate radiomic features that may be able to predict CPP through a ML algorithms. We identified two different radiomic parameters, Shape Surface Volume Ratio and Glrlm Gray Level Non-Uniformity, which, through the application of our ML algorithm, allowed to predict CPP with a high diagnostic accuracy (ROC-AUC 0.81 ± 0.08). We confirmed also a diagnostic value of PV (ellipsoid formula), however, this reference model had a much lower sensitivity, in accordance with previous studies (17). In addition, radiomics showed a high inter-reader reliability. Clinical and anthropometric variables were not confounding factors of the main RFs of interest suggesting that premature thelarche and/or pubarche would not be potentially misclassified versus CPP. Finally, basal and peak gonadotropin levels were correlated with the selected RFs. Very few studies to date have investigated the role of radiomics in the diagnostic workup of CPP. Jiang et al. (42) developed a radiomic score that was able to predict CPP with a slightly lower performance compared to our model (ROC-AUC = 0.76). However, their patient cohort was very small (18 girls with CPP and 12 healthy controls), and their data showed a huge variability. Zou et al. more recently developed machine learning models based on MRI radiomics and on clinical, hormonal and pelvic ultrasound data to discriminate central precocious puberty from peripheral precocious puberty (43). They obtained promising results (ROC-AUC = 0.86) from the integration of multiomic/multimodal data, while less optimal findings (ROC-AUC = 0.67) for radiomics alone. However, these results also are not completely comparable with our findings due to the different target of the predictive models. Therefore, to the best of our knowledge until now, the Literature lacks reliable data highlighting the ability of MRI radiomics to differentiate between CPP patients and control cases.

This study provides new meaning to MRI findings, and ideally could lead to a significant change in the diagnostic assessment of CPP, without the need for a GnRH stimulation test.

The determination of basal LH alone is not currently considered sufficient, although discussed, for the diagnosis of CPP (4) but could potentially become such in addition to “potentiated” MRI imaging of the pituitary gland without the need for further testing. Over the past years in fact, several studies have investigated the accuracy of baseline gonadotropins in the diagnostic workup of CPP. Some studies have suggested that LH values <0.2 IU/L are associated with a lower risk of pubertal activation, while LH >1 IU/L generally would display a high positive predictive value. However, precise reference values have not yet been identified, both for the confirmation or the exclusion of CPP, hence the need to perform a GnRH test in many cases (5, 44, 45). In our radiomic model, interestingly, peak LH did not provide additional information with respect to basal LH.

The radiomic model reflected the changes that the pituitary gland undergoes during puberty related with the proliferation of specific cell populations within and increased secretion of hormones (46, 47). During adolescence, in humans, the pituitary gland shows a “growth spurt” with increase in volume and height that is generally greater in girls than in boys, as a reflection of the activation of different hormonal profiles (48, 49). Therefore, the association we found between the radiomic parameter Shape Surface Volume Ratio with basal and peak LH values may underline this functional-morphological correlation. Moreover, the correlation of both basal and peak LH with the non-uniformity parameters (Glrlm Gray Level Non-Uniformity) highlight once again how radiomics reflects functional aspects (43).

The method has anyway limitations, as shown by the misclassification of case n. 15 suggesting that it might not be accurate in subjects having very small pituitary volumes, and at the very initial stages of activation. This could be mitigated, however, using a high-resolution MRI sequence, and is justified also by the existence of a minority of CPP cases that had borderline morphological and functional characteristics. Furthermore, the main limitation of this study is represented by the small number of subjects and further validation studies are warranted. Finally, we showed good reliability of findings using both an unexperienced, and a moderately experienced reader.

Concluding, these data open the way to the potential use of radiomics for the diagnosis of CPP.

## Data Availability

The original contributions presented in the study are included in the article/**Supplementary Material**, further inquiries can be directed to the corresponding author. Requests to access the dataset should be directed to mariaelisabeth.street@unipr.it.
