# Supplementary material for: Magnetic resonance imaging -based radiomics of the pituitary gland is highly predictive of precocious puberty in girls: a pilot study
Source: Front Endocrinol (Lausanne). 2025 Feb 5;16:1496554. doi: 10.3389/fendo.2025.1496554 (PMC11835667; doi:10.3389/fendo.2025.1496554)
Supplement: Supplementary file 1 [file DataSheet1.docx]

Supplementary Material

# Supplementary Figure

## Supplementary Figure 1. Correlation between pituitary volume measurements. Horizontal axis: ellipsoid method. Vertical axis: radiomics.





# Supplementary Tables

## Supplementary Table 1. Significant associations between RFs (also those not included in radiomic model) and categorical endpoint surrogates describing the clinical stage of puberty. *Significant difference (p-value < 0.05) of RF value among endpoint (surrogate) subgroups in at least 50% of rounds.

| **Endpoint surrogate** | **RFs significantly associated with the categorical endpoint surrogate*** |
| --- | --- |
| Tanner Breast stage | \| gldmDependenceVariance \| \| --- \| \| glrlmRunVariance \| \| gldmDependenceNonUniformityNormalized \| \| glrlmLongRunEmphasis \| \| glcmMaximumProbability \| \| gldmLargeDependenceEmphasis \| \| glrlmRunPercentage \| \| glcmIdm \| \| glrlmRunLengthNonUniformityNormalized \| \| glcmId \| \| glcmJointEnergy \| \| glrlmShortRunEmphasis \| \| gldmGrayLevelNonUniformity \| \| glcmJointEntropy \| \| firstorderInterquartileRange \| \| firstorderRobustMeanAbsoluteDeviation \| \| firstorderUniformity \| \| **Volume: ellipsoid method** \| |
| Tanner Pubic Hair stage | \| ngtdmStrenght \| \| --- \| \| glszmGrayLevelNonUniformityNormalized \| \| glszmLargeAreaLowGrayLevelEmphasis \| \| ngtdmBusyness \| \| glszmZoneVariance \| \| glszmZonePercentage \| \| glszmLargeAreaEmphasis \| \| ngtdmComplexity \| \| gldmSmallDependenceEmphasis \| \| gldmGrayLevelNonUniformity \| \| glszmGrayLevelVariance \| |

## Supplementary Table 2. Significant correlations between RFs (also those included in radiomic model) and hormonal and pelvic US endpoints. *Correlation with p-value < 0.05 in at least 50% of rounds. ^The size of correlation was attributed according to the mean value of the correlation coefficient (R ̅) across 100 rounds.

| **Endpoint surrogates** | **RFs significantly correlated *** | **Size of correlation ^** |  |  |  |  |  |
| --- | --- | --- | --- | --- | --- | --- | --- |
| basal LH | \| gldmDependenceNonUniformityNormalized \| \| --- \| \| gldmGrayLevelNonUniformity \| \| **glrlmGrayLevelNonUniformity** \| \| glszmLargeAreaEmphasis \| \| glszmLargeAreaHighGrayLevelEmphasis \| \| glszmZoneVariance \| \| ngtdmCoarseness \| \| ngtdmStrenght \| \| shapeLeastAxisLength \| \| shapeMaximum2DDiameterSlice \| \| shapeMeshVolume \| \| shapeMinorAxisLength \| \| s**hapeSurfaceVolumeRatio** \| \| shapeVoxelVolume \| \| glrlmLongRunEmphasis \| \| shapeSurfaceArea \| \| glrlmRunPercentage \| \| gldmLargeDependenceEmphasis \| \| glrlmRunLengthNonUniformityNormalized \| \| gldmDependenceVariance \| \| glszmZonePercentage \| \| gldmSmallDependenceEmphasis \| \| glrlmRunVariance \| \| shapeElongation \| \| shapeFlatness \| \| glcmImc2 \| \| glrlmShortRunEmphasis \| \| **Volume: ellipsoid method** \| | \| WEAK \| \| --- \| \| MODERATE \| \| **MODERATE** \| \| WEAK \| \| MODERATE \| \| WEAK \| \| WEAK \| \| WEAK \| \| WEAK \| \| MODERATE \| \| MODERATE \| \| MODERATE \| \| **WEAK** \| \| MODERATE \| \| WEAK \| \| WEAK \| \| WEAK \| \| WEAK \| \| WEAK \| \| WEAK \| \| WEAK \| \| WEAK \| \| WEAK \| \| WEAK \| \| WEAK \| \| WEAK \| \| WEAK \| \| **MODERATE** \| |  |  |  |  |  |
| peak LH | \| gldmGrayLevelNonUniformity \| \| --- \| \| **glrlmGrayLevelNonUniformity** \| \| glszmLargeAreaHighGrayLevelEmphasis \| \| glszmZoneVariance \| \| ngtdmCoarseness \| \| shapeLeastAxisLength \| \| shapeMaximum2DDiameterSlice \| \| shapeMeshVolume \| \| shapeMinorAxisLength \| \| shapeSurfaceArea \| \| **shapeSurfaceVolumeRatio** \| \| shapeVoxelVolume \| | \| WEAK \| \| --- \| \| **WEAK** \| \| MODERATE \| \| WEAK \| \| WEAK \| \| WEAK \| \| WEAK \| \| MODERATE \| \| MODERATE \| \| WEAK \| \| **WEAK** \| \| MODERATE \| |  |  |  |  |  |
| basal FSH | \| glcmImc1 \| \| --- \| \| shapeFlatness \| \| glcmMCC \| \| glcmImc2 \| \| shapeElongation \| \| **Volume: ellipsoid method** \| | \| WEAK \| \| --- \| \| WEAK \| \| WEAK \| \| WEAK \| \| WEAK \| \| **WEAK** \| |  |  |  |  |  |
| peak FSH | | \| shapeLeastAxisLength \| \| --- \| | \| WEAK \| \| --- \| |  |  |  |  |
| Left ovarian volume | | | \| shapeMaximum2DDiameterSlice \| \| --- \| \| shapeMinorAxisLength \| \| shapeSurfaceArea \| \| shapeVoxelVolume \| \| shapeMeshVolume \| \| **glrlmGrayLevelNonUniformity** \| \| glszmSizeZoneNonUniformityNormalized \| | \| MODERATE \| \| --- \| \| MODERATE \| \| WEAK \| \| WEAK \| \| WEAK \| \| **WEAK** \| \| WEAK \| |  |  |  |
| Right ovarian volume | | | | \| shapeMinorAxisLength \| \| --- \| \| glszmSizeZoneNonUniformityNormalized \| | \| WEAK \| \| --- \| \| WEAK \| |  |  |
| Uterine length | | | | | \| shapeMajorAxisLength \| \| --- \| | \| MODERATE \| \| --- \| \|  \| |  |
| Fundus/cervix ratio | | | | | None |  |  |
| Estradiol | | | | | | \| ngtdmContrast \| \| --- \| \| gldmLargeDependenceHighGrayLevelEmphasis \| \| glcmIdmn \| \| gldmDependenceNonUniformityNormalized \| \| shapeMaximum2DDiameterSlice \| \| glszmLargeAreaHighGrayLevelEmphasis \| | \| WEAK \| \| --- \| \| WEAK \| \| WEAK \| \| WEAK \| \| WEAK \| \| WEAK \| |

## Supplementary Table 3. Reliability of radiomic features and of the ellipsoid evaluation of pituitary volume.

| **Predictor** | **icc2** | **p-value** | **Predictor** | **icc2** | **p-value** |
| --- | --- | --- | --- | --- | --- |
| volume: ellipsoid method | 0.47 | 6.0E-06 | glcmSumAverage | 0.45 | 3.7E-06 |
| shapeElongation | 0.50 | 1.8E-07 | glcmSumEntropy | 0.57 | 1.0E-09 |
| shapeFlatness | 0.53 | 2.5E-08 | glcmSumSquares | 0.62 | 2.1E-11 |
| shapeLeastAxisLength | 0.56 | 5.0E-05 | gldmDependenceEntropy | 0.00 | 4.9E-01 |
| shapeMajorAxisLength | 0.47 | 8.3E-07 | gldmDependenceNonUniformity | 0.56 | 7.4E-07 |
| shapeMaximum2DDiameterColumn | 0.50 | 1.0E-07 | gldmDependenceNonUniformityNormalized | 0.67 | 1.4E-13 |
| shapeMaximum2DDiameterRow | 0.48 | 4.3E-07 | gldmDependenceVariance | 0.41 | 3.1E-05 |
| shapeMaximum2DDiameterSlice | 0.29 | 3.0E-03 | gldmGrayLevelNonUniformity | 0.47 | 9.9E-07 |
| shapeMaximum3DDiameter | 0.38 | 1.1E-04 | gldmGrayLevelVariance | 0.61 | 5.6E-11 |
| shapeMeshVolume | 0.59 | 1.3E-05 | gldmHighGrayLevelEmphasis | 0.47 | 1.1E-06 |
| shapeMinorAxisLength | 0.61 | 1.5E-10 | gldmLargeDependenceEmphasis | 0.40 | 3.6E-05 |
| shapeSphericity | 0.57 | 1.1E-09 | gldmLargeDependenceHighGrayLevelEmphasis | 0.34 | 5.1E-04 |
| shapeSurfaceArea | 0.58 | 3.6E-05 | gldmLargeDependenceLowGrayLevelEmphasis | 0.30 | 2.1E-03 |
| shapeSurfaceVolumeRatio | 0.68 | 2.1E-10 | gldmLowGrayLevelEmphasis | 0.21 | 2.3E-02 |
| shapeVoxelVolume | 0.59 | 1.5E-05 | gldmSmallDependenceEmphasis | 0.54 | 1.6E-08 |
| firstorder10Percentile | 0.58 | 4.5E-10 | gldmSmallDependenceHighGrayLevelEmphasis | 0.46 | 1.6E-06 |
| firstorder90Percentile | 0.59 | 2.3E-10 | gldmSmallDependenceLowGrayLevelEmphasis | 0.09 | 2.0E-01 |
| firstorderEnergy | 0.59 | 2.0E-10 | glrlmGrayLevelNonUniformity | 0.58 | 1.1E-09 |
| firstorderEntropy | 0.56 | 2.0E-09 | glrlmGrayLevelNonUniformityNormalized | 0.55 | 3.8E-09 |
| firstorderInterquartileRange | 0.59 | 2.8E-10 | glrlmGrayLevelVariance | 0.60 | 1.2E-10 |
| firstorderKurtosis | 0.17 | 3.4E-02 | glrlmHighGrayLevelRunEmphasis | 0.47 | 1.5E-06 |
| firstorderMaximum | 0.54 | 1.4E-08 | glrlmLongRunEmphasis | 0.35 | 3.3E-04 |
| firstorderMeanAbsoluteDeviation | 0.60 | 5.6E-11 | glrlmLongRunHighGrayLevelEmphasis | 0.36 | 1.8E-04 |
| firstorderMean | 0.49 | 3.4E-07 | glrlmLongRunLowGrayLevelEmphasis | 0.27 | 4.5E-03 |
| firstorderMedian | 0.57 | 1.6E-09 | glrlmLowGrayLevelRunEmphasis | 0.22 | 1.8E-02 |
| firstorderMinimum | 0.30 | 2.0E-03 | glrlmRunEntropy | 0.64 | 2.6E-12 |
| firstorderRange | 0.50 | 1.8E-07 | glrlmRunLengthNonUniformity | 0.49 | 4.5E-06 |
| firstorderRobustMeanAbsoluteDeviation | 0.62 | 1.3E-11 | glrlmRunLengthNonUniformityNormalized | 0.51 | 7.9E-08 |
| firstorderRootMeanSquared | 0.58 | 7.1E-10 | glrlmRunPercentage | 0.47 | 1.0E-06 |
| firstorderSkewness | 0.22 | 1.5E-02 | glrlmRunVariance | 0.33 | 6.0E-04 |
| firstorderTotalEnergy | 0.58 | 8.8E-10 | glrlmShortRunEmphasis | 0.48 | 5.5E-07 |
| firstorderUniformity | 0.53 | 2.1E-08 | glrlmShortRunHighGrayLevelEmphasis | 0.48 | 6.6E-07 |
| firstorderVariance | 0.61 | 6.3E-11 | glrlmShortRunLowGrayLevelEmphasis | 0.19 | 3.9E-02 |
| glcmAutocorrelation | 0.47 | 8.8E-07 | glszmGrayLevelNonUniformity | 0.37 | 2.5E-03 |
| glcmClusterProminence | 0.43 | 5.7E-06 | glszmGrayLevelNonUniformityNormalized | 0.25 | 6.8E-03 |
| glcmClusterShade | 0.42 | 1.3E-05 | glszmGrayLevelVariance | 0.12 | 1.3E-01 |
| glcmClusterTendency | 0.63 | 9.7E-12 | glszmHighGrayLevelZoneEmphasis | 0.00 | 5.0E-01 |
| glcmContrast | 0.37 | 1.1E-04 | glszmLargeAreaEmphasis | 0.39 | 5.5E-05 |
| glcmCorrelation | 0.71 | 5.8E-16 | glszmLargeAreaHighGrayLevelEmphasis | 0.43 | 7.2E-06 |
| glcmDifferenceAverage | 0.58 | 4.4E-10 | glszmLargeAreaLowGrayLevelEmphasis | 0.25 | 7.9E-03 |
| glcmDifferenceEntropy | 0.56 | 2.6E-09 | glszmLowGrayLevelZoneEmphasis | 0.20 | 2.8E-02 |
| glcmDifferenceVariance | 0.57 | 9.2E-10 | glszmSizeZoneNonUniformity | 0.36 | 1.7E-04 |
| glcmId | 0.53 | 1.6E-08 | glszmSizeZoneNonUniformityNormalized | 0.10 | 1.6E-01 |
| glcmIdm | 0.54 | 7.6E-09 | glszmSmallAreaEmphasis | 0.17 | 4.9E-02 |
| glcmIdmn | 0.20 | 2.1E-02 | glszmSmallAreaHighGrayLevelEmphasis | 0.35 | 3.0E-04 |
| glcmIdn | 0.27 | 3.7E-03 | glszmSmallAreaLowGrayLevelEmphasis | 0.13 | 1.1E-01 |
| glcmImc1 | 0.58 | 8.1E-08 | glszmZoneEntropy | 0.44 | 3.9E-06 |
| glcmImc2 | 0.65 | 5.5E-13 | glszmZonePercentage | 0.61 | 6.3E-11 |
| glcmInverseVariance | 0.42 | 1.8E-05 | glszmZoneVariance | 0.38 | 1.1E-04 |
| glcmJointAverage | 0.45 | 3.7E-06 | ngtdmBusyness | 0.25 | 7.3E-03 |
| glcmJointEnergy | 0.46 | 1.5E-06 | ngtdmCoarseness | 0.48 | 3.6E-06 |
| glcmJointEntropy | 0.55 | 3.4E-09 | ngtdmComplexity | 0.53 | 3.8E-08 |
| glcmMCC | 0.58 | 1.4E-09 | ngtdmContrast | 0.47 | 5.8E-07 |
| glcmMaximumProbability | 0.48 | 5.7E-07 | ngtdmStrenght | 0.45 | 1.1E-05 |
